# A neonatal cluster of novel coronavirus disease 2019: clinical management and considerations

**DOI:** 10.1186/s13052-020-00947-9

**Published:** 2020-12-07

**Authors:** Nicole Olivini, Francesca Ippolita Calò Carducci, Veronica Santilli, Maria Antonietta De Ioris, Alessia Scarselli, Dario Alario, Caterina Geremia, Mary Haywood Lombardi, Caterina Marabotto, Rosanna Mariani, Raffaele Edo Papa, Emanuela Peschiaroli, Raffaella Scrocca, Serena Sinibaldi, Andrea Smarrazzo, Pietro Stella, Stefania Bernardi, Sara Chiurchiù, Paola Pansa, Lorenza Romani, Carletti Michaela, Carlo Concato, Domenico Umberto De Rose, Gugliemo Salvatori, Paolo Rossi, Alberto Villani, Andrea Dotta, Patrizia D’Argenio, Andrea Campana

**Affiliations:** 1 Pediatrics Unit, University Department of Pediatrics (DPUO), Bambino Gesù Children’s Hospital – IRCCS, Via della Torre di Palidoro, 00050 Fiumicino, Rome Italy; 2 Research Unit in Congenital and Perinatal Infection, Immune and Infectious Diseases Division, University Department of Pediatrics (DPUO), Bambino Gesù Children’s Hospital – IRCCS, Rome, Italy; 3 Pediatrics and Neonatology Unit, San Paolo Hospital, Civitavecchia, Italy; 4 Pediatrics and Infectious Diseases Unit, Bambino Gesù Children’s Hospital - IRCCS, Rome, Italy; 5 Laboratory Unit, Bambino Gesù Children’s Hospital – IRCCS, Rome, Italy; 6 Virology Unit, Bambino Gesù Children’s Hospital – IRCCS, Rome, Italy; 7 Department of Neonatology, Bambino Gesù Children’s Hospital – IRCCS, Rome, Italy; 8 Department of Systems Medicine, University of Rome Tor Vergata, Rome, Italy

**Keywords:** SARS-CoV-2, COVID-19, Newborns, Pandemic, Infection

## Abstract

**Background:**

Lately, one of the major clinical and public health issues has been represented by Coronavirus disease of 2019 (COVID-19) during pregnancy and the risk of transmission of the infection from mother to child. Debate on perinatal management and postnatal care is still ongoing, principally questioning the option of the joint management of mother and child after birth and the safety of breastfeeding. According to the available reports, neonatal COVID-19 appears to have a horizontal transmission and seems to be paucisymptomatic or asymptomatic, compared to older age groups. The aim of this work is to describe a cluster of neonatal COVID-19 and discuss our experience, with reference to current evidence on postnatal care and perinatal management.

**Methods:**

This is a retrospective observational case series of five mother-child dyads, who attended the Labor and Delivery Unit of a first-level hospital in Italy, in March 2020. Descriptive statistics for continuous variables consisted of number of observations, mean and the range of the minimum and maximum values.

**Results:**

Five women and four neonates tested positive for Severe Acute Respiratory Syndrome Coronavirus 2 (SARS-CoV-2). In one case, the mother-child dyad was separated and the neonate remained negative on two consecutive tests. Two positive neonates developed symptoms, with a predominant involvement of the gastrointestinal tract. Blood tests were unremarkable, except for a single patient who developed mild neutropenia. No complications occurred.

**Conclusions:**

We agree that the decision on whether or not to separate a positive/suspected mother from her child should be made on an individual basis, taking into account the parent’s will, clinical condition, hospital logistics and the local epidemiological situation. In conformity with literature, in our study, affected neonates were asymptomatic or paucisymptomatic. Despite these reassuring findings, a few cases of severe presentation in the neonatal population have been reported. Therefore, we agree on encouraging clinicians to monitor the neonates with a suspected or confirmed infection.

## Introduction

In November 2019, the first case of Severe Acute Respiratory Syndrome Coronavirus 2 (SARS-CoV-2) infection was reported in China [1]. Since then, the novel coronavirus disease of 2019 (COVID-19) has rapidly progressed to a global pandemic. Due to its rapid rate of transmission, 49,727,316 confirmed cases of COVID-19 have been reported globally as of November 08, 2020, with 1,248,373 deaths [2]. Italy, among the Nations first hit by the pandemic, accounts for 558,636 confirmed cases and 41,394 deaths [3].

One of the major clinical and public health issues has been represented by COVID-19 during pregnancy and the risk of transmission of the infection from mother to child before, during and after delivery [4–7]. Debate on perinatal management and postnatal care is still ongoing, principally questioning the option of the joint management of mother and child after birth and the safety of breastfeeding [8–14]. Based on available literature, neonatal COVID-19 appears to have a horizontal transmission and seems to be paucisymptomatic or asymptomatic, compared to older age groups, similarly to the 2002–2003 SARS-CoV-1 epidemic [15–20].

In this work, we describe a cluster of neonatal novel Coronavirus infection. We furthermore discuss our experience on clinical and management aspects, with reference to currently available evidence.

## Materials and methods

This is a retrospective observational case series of five mother-child dyads. Informed consent was obtained from each patient and the protocol conforms to the ethical guidelines of the 1975 Declaration of Helsinki, as reflected in a priori approval by the Institution’s Human Research Committee.

The patients’ clinical records were reviewed for data including demographic information, contact history, prenatal history, clinical symptoms, routine laboratory tests and virology.

Complete blood count and chem-panel were run by our internal Clinical Biochemistry Laboratory on samples collected during routine clinical blood draws. The *Bambino Gesù* Laboratory of Virology took care of the detection of SARS-CoV-2 on rhino-pharyngeal, rectal swabs and breast milk, through Real Time Polymerase Chain Reaction (RT-PCR) by the mean of established methods [21].

Descriptive statistics for continuous variables consisted of number of observations, mean and the range of the minimum and maximum values, median and interquartile range (IQR).

## Results

### Cluster description

In early March 2020, a puerpera in the Labor and Delivery Unit of a first-level hospital in the province of Rome was confirmed positive for SARS-CoV-2 on rhino-pharyngeal swabs. She had been tested on admission, on account of close exposure to an infected person (her partner). On day 2, she gave birth to a healthy baby boy through caesarean section, without complications. Precautionary measures were adopted for both the mother and the child, in agreement between the health providers and the mother herself, who had been previously informed on the possible options of maternal and perinatal care, according to currently available recommendations. After birth, the neonate was immediately examined by a pediatric team, transported in a closed crib and isolated in a dedicated area of the hospital nursery. An oropharyngeal swab specimen, obtained 24 h later, showed a negative result for SARS-CoV-2. Twenty-four hours later, the infant’s rhino-pharyngeal swab confirmed negative and precautionary isolation in the hospital nursery was suspended, even though separation from the mother persisted. Expressed breast milk was regularly offered to the baby, following careful application of preventive procedures, such as wearing a mask and cleaning the breasts and hands of the mother before pumping. The neonate remained healthy throughout the stay in the hospital nursery. He was discharged 7 days after birth and entrusted to the care of his grandparents, in accordance with the indications of the local health authorities, since his mother and father were both positive for SARS-CoV-2 upon discharge. Expressed maternal breast milk after appropriate hygiene was offered to the neonate by the healthy grandparents.

Ten days later, a nurse of the same first-level hospital in the province of Rome tested positive for SARS-CoV-2. She had been in contact with as many as 27 neonates and their mothers in the two weeks preceding the result of her test and had assisted the puerpera who resulted infected in early March. Exposed neonates and mothers were subsequently recalled by the local health authorities and tested for the virus. A total of 54 rhino-pharyngeal swabs were obtained (27 neonates, 27 mothers). Among these, 4 neonates and their respective mothers tested positive and were admitted to the COVID-19 Center of *Bambino Gesù* Children’s Research Hospital of Palidoro (Rome) for surveillance and care.

### Maternal characteristics

A total of 5 women who attended the Labor and Delivery Unit of the same first-level hospital in the province of Rome tested positive for SARS-CoV-2, at different times. Mean maternal age was 29.8 years (range 24–36). No co-morbidities and no complications during pregnancy have been described. Cigarette smoking was reported as a maternal risk factor once. Two women gave birth through vaginal delivery; 3 had a planned elective caesarean section. Shoulder dystocia occurred once, while the other deliveries were uneventful.

Three women reported symptoms: 1 low grade fever, 2 anosmia, 1 dysgeusia, 1 musculoskeletal pain. Onset of symptoms occurred at day 1, 5 and 19 after childbirth. No significant laboratory test abnormalities were detected. Women did not receive any antiviral and immunomodulating therapy.

Four out of 5 mothers directly breastfed their babies, without any precautionary measure, being unaware of their exposure history. Among them, 2 mothers, after the diagnosis of COVID-19 and by personal choice, stopped breastfeeding and offered their babies formula milk. At the COVID-19 Center of *Bambino Gesù* Children’s Research Hospital of Palidoro (Rome), RT-PCR for detecting SARS-CoV-2 in breast milk was performed for those mothers who were still breastfeeding. The two samples of expressed milk collected from *Mother 2* and *Mother 4* tested negative for the presence of SARS-CoV-2 (Table 1).

**Table 1:** Maternal characteristics, clinical and virological findings

Mother ID	Age years	Symptoms	Symptoms onset days from delivery	1st RPS (days from delivery)	2nd RPS (days from delivery)	3rd RPS (days from delivery)	4th RPS (days from delivery)	5th RPS (days from delivery)	SARS-CoV-2 on breast milk (days from delivery)
1	24	No	–	**POS** (−1)	**POS** (+ 2)	**POS** (+ 19)	**POS** (+ 32)	–	–
2	36	Anosmia, dysgeusia	+ 19	**POS** (+ 16)	**POS** (+ 20)	**POS** (+ 23)	**POS** (+ 26)	**POS** (+ 29)	NEG (+ 23)
3	27	No	–	**POS** (+ 12)	NEG (+ 15)	NEG (+ 17)	–	–	
4	26	Anosmia, musculo-skeletal pain	+ 5	**POS** (+ 6)	NEG (+ 10)	**POS** (+ 11)	**POS** (+ 13)	**POS** (+ 17)	NEG (+ 13)
5	36	Low grade fever	+ 1	**POS** (+ 4)	NEG (+ 9)	NEG (+ 11)	–	–	

***RPS*** rhino-pharyngeal swab, ***SARS-CoV-2*** severe acute respiratory syndrome coronavirus 2, ***RT-PCR*** real time polymerase chain reaction, ***POS*** positive result, ***NEG*** negative result

### Neonatal characteristics

A total of 5 neonates born to positive mothers were tested. Four neonates (2 females, 2 males) resulted positive for SARS-CoV-2. Among them, 3 were born at term and 1 was late preterm. All of the 4 positive newborn infants were roomed-in in their mothers’ rooms and breastfed.

Deliveries and perinatal history were uneventful for 2 of them. *Neonate 2* had complicated shoulder dystocia resulting in perinatal depression. He was given oxygen support through positive pressure ventilation and recovered within 30 s of primary resuscitation procedures. Brachial plexus injury was noticed right after childbirth and addressed to specialist care. X-rays ruled out a clavicle fracture. At the time of this writing, the infant is 3 months old, has partially recovered with physical therapy and has been following regular follow up in our Rehab Center. *Neonate 3* was born through meconium-stained amniotic fluid, but she was vigorous at birth and stay healthy throughout the hospital stay.

Two of the positive neonates developed symptoms possibly related to SARS-CoV-2. *Neonate 4* showed frequent regurgitation and subsequent diarrhea from her 5th day of life. When she was admitted to the COVID-19 Center, supportive fluid therapy was begun for mild dehydration. Complete resolution of diarrhea occurred 7 days later, but the neonate was kept for observation because of poor growth, which was attributed to maternal hypogalactia, at last. Microbiological and virological examinations performed on stool samples did not identify any possibly responsible pathogen. *Neonate 5* developed irritability and feeding difficulties within 24 h from birth; his blood tests showed a raised leukocyte count and high levels of inflammatory markers. Antibiotics (ampicillin 150 mg/kg/die, q8h and gentamycin 5 mg/kg/die, q24h) were promptly started and maintained for a total of 6 days, with clinical benefit and complete normalization of blood tests. All cultures were negative. The newborn baby was transferred to the COVID-19 Center of *Bambino Gesù* Children’s Research Hospital of Palidoro (Rome) on his 8th day of life.

Routine laboratory tests were unremarkable in three patients; *Neonate 2* developed mild neutropenia (minimum 800 cell/mmc) during hospital stay, which resolved spontaneously 7 days later (Table 2).

**Table 2:** Neonatal history, general characteristics and clinical and virological findings during hospital stay at the COVID-19 Center of *Bambino Gesù* Children’s Hospital of Palidoro

Neonate ID	Gender	Gestational Age	Delivery	AS 1′/5’	Birth Weight (grams)	Maternal Risk Factors	Perinatal Problems	Bonding	Breastfeeding			Rooming-in	Age at Nursery Discharge (days)			
1	M	41 ^+ 0^	CS (transverse lie)	9/10	3900	No	No	No	Expressed milk			No	7			
2	M	41 ^+ 2^	V	5/10	4440	No	Shoulder dystocia	Yes	Direct			Yes	3			
3	F	36 ^+ 0^	V	9/10	2760	No	MSAF, vigorous	Yes	Direct, formula			Yes	3			
4	F	38 ^+ 6^	CS	9/10	3120	No	No	Yes	Direct			Yes	4			
5	M	39 ^+ 0^	CS (podalic fetus)	9/10	2750	Smoke	Congenital anomalies of the GUT, perinatal infection	Yes	Direct, formula			Yes	8 (transferred)			
							RHINOPHARYNGEAL SWABS						RECTAL SWABS			
Neonate ID	**Age on Admission** (days)	**Age on discharge** (days)	**Symptoms**	**Age at Symptoms Onset** (days)	**Abnormal Lab Tests**	**Therapy**	**1st** (age in days)	**2nd** (age in days)	**3rd** (age in days)	**4th** (age in days)	**5th** (age in days)	**6th** (age in days)	**1st** (age in days)	**2nd** (age in days)	**3rd** (age in days)	**4th** (age in days)
1^a^	–	–	No	–	–	–	NEG (2)	NEG (3)	–	–	–	–	–	–	–	–
2	19	–	No	–	Neutropenia	No	**POS** (16)	**POS** (20)	IND (23)	**POS** (26)	IND (29)	NEG (32/35)	**POS** (20)	**POS** (23)	**POS** (26)	**POS** (29)
3	14	19	No	–	No	No	**POS** (12)	NEG (14)	NEG (15)	NEG (17)	–	–	NEG (15)	NEG (17)	–	–
4	10	–	Cough, diarrhea, regurgitation	5	–	Fluids	**POS** (6)	NEG (10)	NEG (11)	NEG (13)	NEG (17)	NEG (20)	NEG (11)	NEG (13)	NEG (17)	NEG (20)
5	8	13	Irritability, feeding difficulties	0–1	Neutrophilia high PCT and CRP	Antibiotic treatment	**POS** (4)	NEG (9)	NEG (11)	–	–	–	NEG (9)	NEG (11)	–	–

***PCT*** procalcitonin, ***CRP*** C-reactive protein

^a^ Neonate 1 was not admitted to the COVID-19 Center of *Bambino Gesù* Children’s Hospital of Palidoro because of negative results. We include him for the sake of completeness

***CS*** caesarean section, ***V*** vaginal delivery, ***AS*** Apgar score, ***MSAF*** meconium stained amniotic fluid, ***GUT*** genitourinary tract

At the COVID-19 Center of *Bambino Gesù* Children’s Research Hospital of Palidoro (Rome), SARS-CoV-2 RT-PCR on rhino-pharyngeal and rectal swabs was performed on admission and every 48–72 h, until two consecutive negative results.

The mean time to negative rhino-pharyngeal swab from the first positive result was 6.75 days (range 2–16); with a median value of 4.5 (IQR 3.5; 7.75). Compared to other patients, *Neonate 2* showed a prolonged SARS-CoV-2 RNA detection on both rhino-pharyngeal and rectal swabs. *Neonates 3–5* had always had negative results on rectal swabs.

Mean and median duration of hospital stay were respectively 12.5 days (range 5–21) and 12 days (IQR 5;19.5); all neonates were discharged home in good clinical conditions (Table 2).

Telephone follow-up was performed on a regular basis up to a month from hospital discharge, to monitor the recovery period and eventually identify at an early stage potential recrudescence or a second phase of the disease. Parents always reported of their children’s adequate growth and good health. Psychological support was offered to the mother of *Neonate 2*, who experienced symptoms of depression and anxiety.

## Discussion

Perinatal management and postnatal care of infants born to mothers with a suspected or confirmed SARS-CoV-2 infection and the clinical characteristics of COVID-19 in newborns, infants and children are highly relevant topics. According to existing literature, neonatal and pediatric cases are principally family cluster cases; most of them have epidemiological links to adult subjects, but show milder clinical manifestations, with a good prognosis [22].

Since the SARS-CoV-2 outbreak began, scientists have been trying to explain why children are much less likely than adults to show severe presentation and develop complications from the infection. Some hypotheses have been made, including mal-adapting immune response in the elderly compared to children, developmental changes in immunity, with a predominant innate response to infectious stimulus in young infants, effects of lung development and ageing, differences in the physiology and anatomy of the respiratory tract and the crucial role of comorbidities on outcomes [23]. Also, according to some researchers, children’s healthier endothelium may protect them from progression to severe/fatal disease [24], on the contrary to what may happen in adults, in whom problems with the endothelium seem to be related with a worse prognosis [25].

In conformity with literature, in our case series affected neonates were asymptomatic or paucisymptomatic [19, 22, 26–28]. Differently from other reports on neonatal and pediatric COVID-19, we did not observe fever, nor respiratory symptoms [19, 29, 30]. In our series, the gastrointestinal tract was predominantly interested, in accordance with other reports [31] and one infant presented failure to thrive. On the neonate who was treated with antibiotic therapy from day 2 to day 8 of life due to irritability, feeding difficulties and high levels of inflammatory markers (*Neonate 5*), blood cultures turned negative, leaving the question open to whether his symptoms could be ascribed to a neonatal early-onset bacterial infection or to a systemic viral disease.

Altered laboratory tests have also been reported [17–19]. Only a patient in our case series developed blood tests abnormalities during hospital stay, consisting in mild neutropenia, possibly due to different pathophysiological mechanisms related to systemic viral infection, including inhibition of hematopoiesis, granulocyte sequestration, margination, and peripheral destruction [32]. Neutropenia in neonates affected with COVID-19 was also reported in previous case series and reviews [30, 33, 34]. No leukocytopenia nor lymphocytopenia have been observed among our patients, although described in previous studies [17, 18, 26].

Regarding clinical presentation and outcomes, although the majority of reports and our experience described mild symptoms and good outcomes, a few cases of moderate-to-severe presentation and death of neonates born to mothers with COVID-19 have been observed [19, 28, 33]. Thus, we agree on encouraging caregivers of neonates to adopt preventive measures when coming into contact with neonates, in order to limit community-spread of SARS-CoV-2 to this potentially vulnerable population. Additionally, we agree on closely monitor the neonates with a suspected or confirmed SARS-CoV-2 infection [28, 33].

This pandemic is a rapidly growing challenge for labor and delivery units, as long as for hospital nurseries and neonatal care units. There is still an ongoing debate as to whether neonates should be isolated from mothers suspected or confirmed positive for SARS-CoV-2 infection and whether direct breastfeeding is considered to be safe. Significantly different positions have been adopted by international Health Organizations, so far [4, 9, 18, 35–40]. According to the Italian National Institute of Health (Istituto Superiore di Sanità, ISS), the Italian Society of Neonatology (SIN) and the Union of European Neonatal & Perinatal Societies (UENPS), a woman with a suspected or confirmed COVID-19, under favorable clinical conditions and according to her desire, should start and continue to breastfeed, directly to the breast or using non-pasteurized expressed breast milk, applying preventive procedures such as hand hygiene and the use of a face mask during feeds. With regards to whether or not to separate mother and child, the ISS suggests the decision should be made on an individual basis, taking into account the informed consent of the mother, her clinical conditions, the hospital logistics and possibly the local epidemiological situation relating to the spread of SARS-CoV-2 [14, 41–43]. In our opinion, this approach opens up to a patient-centered, personalized care, by emphasizing the importance of parental will and participation, which must be supported and guided by an exhaustive set of information from the medical staff. Adopting this criterion, a medical team of Pediatricians and Gynecologists together with *Mother 1* decided to manage separately the woman and her newborn and to feed the baby with fresh expressed milk. As previously described, the neonate did well and tested negative for SARS-CoV-2.

Based on current knowledge, the breast milk of a COVID-19 mother cannot be considered a transmission vehicle, similarly to other known respiratory viral infections [13, 44]. In our experience, two mothers, after the diagnosis of COVID-19, personally chose to stop breastfeeding and gave their babies formula. RT-PCR on milk of the breastfeeding mothers tested negative for SARS-CoV-2, in line with previous reports that support breastfeeding [7, 11–13, 44–46]. Moreover, as suggested by Davanzo et al. [45], specific maternal SARS-CoV-2 antibodies pass via the breast milk from the COVID-19 mother to her child within a few days after the onset of the disease, thus possibly modulating the clinical expression of the infant’s infection. Of note, we report the finding of Kirstman et al., who isolated SARS-CoV-2 RNA on a sample of breast milk, collected from a positive mother two days after childbirth. However, as the Authors themselves point out, the potential for respiratory secretion contamination of breast milk could not be ruled out, even though it was minimized by breast hygiene before specimen collection [34].

We did not suggest precautionary measures for the breastfeeding mothers, nor isolation measures, considering that both the mothers and their children were positive on admission.

On mother-to-child transmission modalities, the issue is still controversial. Literature with supporting evidence for vertical transmission and studies on transplacental transmission correlations are still limited [44, 47]. Cases of suspected perinatal infection have been reported [34, 47–49]. Two different research teams in China described 3 neonates, born to mothers with COVID-19, whose IgM antibodies for SARS-CoV-2 following birth were elevated [48, 49]. However, none of these babies had a positive RT-PCR test result, thus not supporting the serologic suggestion of in utero transmission. Also, IgM assays are subject to false-positive and false-negative results, along with cross-reactivity. Kirtsman et al. reported a case of probable congenital SARS-CoV-2 infection, with a positive maternal nasopharyngeal swab two days before childbirth and placental swabs (on both the maternal and fetal side), a vaginal swab and a neonatal rhinopharyngeal swab showing a positive result for SARS-CoV-2 on the day of birth [34]. Recently, Patanè et al. described the presence of SARS-CoV-2 RNA on the fetal side of the placenta in 2 cases of mothers who were diagnosed with COVID-19 and whose neonates had positive test results for SARS-CoV-2 at birth [47]. Even though these findings support the possibility of vertical transmission of SARS-CoV-2, further studies are required to confirm these results [45, 47, 50].

Pediatric cases, as long as older patients, usually have a history of epidemiological exposure [22, 30]. Considering the timing of domiciliary testing and the fact that family clusters or epidemiological links to other infected adults cannot be completely excluded, we could not derive conclusions on transmission modalities in four out of five patients in our series. Moreover, we are not able to demonstrate whether the infection was transmitted from the mother to her child or vice versa. In our case, if we assume that a healthy mother could be infected by her child, considering the greater disease severity described in adults, preventive measures such as mother and child isolation should be also promoted to protect mothers during puerperium.

Testing for SARS-CoV-2 RNA by reverse transcription polymerase chain reaction (RT-PCR) should be reserved to neonates born to mothers with suspected or confirmed COVID-19 and to those presenting with signs of infection suggestive of COVID-19, without neglecting the possibility of alternative diagnoses [51]. Additionally, we of course suggest testing neonates who came into contact with infectious health care professionals. Time for testing ranges from approximately 24 h of life for both symptomatic and asymptomatic neonates born to mothers with suspected or confirmed COVID-19, to the day of onset of signs. Neonates who meet clinical criteria for discharge do not require the results of SARS-CoV-2 testing for leaving the nursery [51]; home isolation and precautions to prevent transmission should continue until the communication of a negative result. According to the last dispositions of the Italian Ministry of Health, discontinuation of home isolation occurs at least 10 days from the first positive result, with a final negative test before isolation is ended, for asymptomatic cases and at least 10 days from the onset of symptoms (the last 3 days in the absence of symptoms), with a final negative test before isolation is ended, for symptomatic cases [52].

The period of time from the onset of symptoms to the first negative RT-PCR test result is defined as *nucleic acid conversion time* and it has been showed that some COVID-19 adult patients experienced a prolonged nucleic acid conversion time, regardless of the presence of clinical symptoms [53–55]. In our cohort we observed a mean time to negative rhino-pharyngeal swab from the first positive result of 6.75 days (range 2–16), with a median of 4.5 [IQR 3.5; 7.75]. Overall, the median age at the 1st negative result was 12 days [IQR 9.75;18.5]. Notably, we cannot derive conclusions on the period of infectiousness, because neonates were tested at different ages and possibly at a different time course of the disease progression, on account of the exposure history. Most of the patients (75%) had negative SARS-CoV-2 rhinopharyngeal PCR samples within a few days from the diagnosis and persistently negative rectal swabs. Only *Neonate 2* showed a prolonged viral shedding, with persistent positive RT-PCR test results on both rhino-pharyngeal and rectal swabs, collected every 72 h during hospital stay. These findings are in line with previous reports that taken together show a considerable variability of viral shedding, among different patients and when examining different routes [53–58].

For those neonates who tested negative on rhino-pharyngeal swabs on admission, at first, we might have expected a positive rectal swab, due to the described extensive persistence of the virus in the gastrointestinal tract [58]. Due to the low probability of false positive nucleic acid detection tests [59], we speculated that not necessarily the SARS-CoV-2 infection in newborns has to involve the respiratory and gastrointestinal tract simultaneously, as proved by daily clinical experience for other viral illnesses (e.g. Adenovirus gastroenteritis and/or respiratory tract infection). Moreover, a study on 20 serial COVID-19 patients showed that infectious virus was not isolated from stool samples, in spite of high virus RNA concentration. The correlation of RT-PCR positivity in stool with retrieval of live virus from the same samples remains to be further investigated [60].

Our study encompasses some limitations, mainly due to its retrospective, observational nature and to the small sample size.

Although from our data, in line with previous studies, it seems that the disease has a milder presentation in neonates, we believe further standardized, larger studies are needed for a better and comprehensive understanding of the characteristics of COVID-19 in the neonatal population.

## Data Availability

All data generated or analysed during this study are included in this published article.
